# Resveratrol Prevents Ammonia Toxicity in Astroglial Cells

**DOI:** 10.1371/journal.pone.0052164

**Published:** 2012-12-21

**Authors:** Larissa Daniele Bobermin, André Quincozes-Santos, Maria Cristina Guerra, Marina Concli Leite, Diogo Onofre Souza, Carlos-Alberto Gonçalves, Carmem Gottfried

**Affiliations:** Department of Biochemistry, Institute of Basic Health Sciences, Federal University of Rio Grande do Sul, Porto Alegre, Rio Grande do Sul, Brazil; Massachusetts General Hospital/Harvard Medical School, United States of America

## Abstract

Ammonia is implicated as a neurotoxin in brain metabolic disorders associated with hyperammonemia. Acute ammonia toxicity can be mediated by an excitotoxic mechanism, oxidative stress and nitric oxide (NO) production. Astrocytes interact with neurons, providing metabolic support and protecting against oxidative stress and excitotoxicity. Astrocytes also convert excess ammonia and glutamate into glutamine via glutamine synthetase (GS). Resveratrol, a polyphenol found in grapes and red wines, exhibits antioxidant and anti-inflammatory properties and modulates glial functions, such as glutamate metabolism. We investigated the effect of resveratrol on the production of reactive oxygen species (ROS), GS activity, S100B secretion, TNF-α, IL-1β and IL-6 levels in astroglial cells exposed to ammonia. Ammonia induced oxidative stress, decreased GS activity and increased cytokines release, probably by a mechanism dependent on protein kinase A (PKA) and extracellular signal-regulated kinase (ERK) pathways. Resveratrol prevented ammonia toxicity by modulating oxidative stress, glial and inflammatory responses. The ERK and nuclear factor-κB (NF-κB) are involved in the protective effect of resveratrol on cytokines proinflammatory release. In contrast, other antioxidants (e.g., ascorbic acid and trolox) were not effective against hyperammonemia. Thus, resveratrol could be used to protect against ammonia-induced neurotoxicity.

## Introduction

Ammonia is implicated as a neurotoxin in brain metabolic disorders associated with hyperammonemia, including hepatic encephalopathy (HE), a neuropsychiatric syndrome, and deficiencies in enzymes of the urea cycle [1], [2]. In these conditions, ammonia concentrations in brain tissue can rise as high as 5 mM [1]. Acute ammonia neurotoxicity can be mediated by an excitotoxic mechanism involving the glutamatergic system, including elevation of extracellular glutamate content, decreased glutamate transporters, NMDA receptor activation and subsequent increases in intracellular calcium concentration [3], [4], [5], [6], [7]. Metabolic effects of ammonia neurotoxicity include changes in reactive oxygen and nitrogen species (ROS/RNS) levels [8], nitric oxide (NO) metabolism [2], cAMP levels [3], [9], mitogen-activated protein kinase (MAPK) pathway [10], cytoskeleton [11] and astrocyte swelling [12]. Moreover, ammonia toxicity also induces increase in tumor necrosis factor α (TNF-α), Interleukin 1β (IL-1β), which can be associated to ROS production and involve protein kinase A (PKA), extracellular signal-regulated kinase (ERK) and nuclear factor-κB (NF-κB) activation [13]. Increased levels of S100B secretion, a protein often used as an indicator of glial activation or death for several types of brain injury [14], [15], were also observed in astrocytes exposed to ammonia [16].

Astrocytes serve a wide range of adaptive functions in the mammalian central nervous system (CNS). These cells interact with neurons, providing structural, metabolic and trophic support, and they can also play a protective role by releasing neurotrophic factors. In pathological circumstances, however, astrocytes have the potential to induce neuronal dysfunction [17], [18], [19], [20]. Astrocytes play an essential role in protecting neurons against excitotoxicity by taking up excess ammonia and glutamate and converting it into glutamine, using the enzyme glutamine synthetase (GS) (EC 6.3.1.2) [21], [22]. Under conditions of ammonia toxicity, GS activity is decreased [3], [16]. This enzyme is very sensitive to oxidative stress, and it has been hypothesized to play an important role in the pathogenesis of ammonia neurotoxicity [23]. The C6 cell line are widely used to study astrocytic functions such as glutamate metabolism (glutamate uptake, GS activity and glutathione production), S100B secretion, oxidative and inflammatory responses [24], [25], [26], [27], [28], [29], [30].

Antioxidants are substances that delay, prevent or reverse oxidative damage to a target molecule [31]. Resveratrol, found in grapes, berries and red wine, is an important antioxidant with a wide range of biological effects. It inhibits carcinogenesis at multiple stages and displays anti-inflammatory, cardioprotective, neuroprotective and anti-aging activities [32], [33], [34]; however, the mechanisms of these effects are not fully understood. Our group has shown that resveratrol also modulates important glial functions including glutamate uptake, GS activity, and glutathione content [27], [35], [36], [37], [38], [39]. The recent literature show that resveratrol is able to protect astrocytes and neurons against a variety of oxidative insults [40], [41], [42], [43].

We therefore investigated the effects of ammonia exposure on ROS/RNS production, GS activity, S100B secretion, proinflammatory cytokines release and NF-κB levels in astroglial cells. We evaluated the effect of resveratrol and other classical antioxidants (ascorbic acid, N^ω^-nitro-L-arginine methyl ester (L-NAME) and trolox) on ROS production and S100B secretion. The mechanism of the protective effect of resveratrol against ammonia toxicity was also explored.

## Materials and Methods

### Materials

Poly-L-lysine, resveratrol, ascorbic acid, trolox, L-NAME, monoclonal anti-S100B (SH-B1), H-89, PD98059, sodium nitroprusside (SNP), propidium iodide (PI), methylthiazolyldiphenyl-tetrazolium bromide (MTT) and 2′-7′-dichlorofluorescein diacetate (DCFH-DA) were purchased from Sigma (St. Louis, MO, USA). Fetal bovine serum (FBS), Dulbecco’s modified Eagle medium (DMEM) and other materials for cell culture were purchased from Gibco BRL (Carlbad, CA, USA). Polyclonal anti-S100B and anti-rabbit peroxidase were purchased from Dako (Glostrup, Denmark) and Amersham (Buckinghamshire, UK), respectively. All other chemicals were purchased from common commercial suppliers.

### C6 Astroglial Cell Culture

The C6 astroglial cell line was obtained from the American Type Culture Collection (Rockville, MA, USA) and was cultured according to a previously described procedure. The cells were seeded in ﬂasks and cultured in DMEM (pH 7.4) containing 5% FBS, 0.1% amphotericin B and 0.032% gentamicin. Cells were maintained at a temperature of 37°C in an atmosphere of 5% CO_2_/95% air. At log phase, cells were detached from the culture ﬂasks using 0.05% trypsin/ethylenediaminetetracetic acid (EDTA) and seeded (5×10^3^ cells/cm^2^) in 96-, 24- or 6-well plates.

### Primary Astrocyte Cell Culture

Primary cortical astrocyte cultures from Wistar rats were prepared as previously described [44]. All procedures were in accordance with the National Institutes of Health Guide for the Care and Use of Laboratory Animals and were approved by the Federal University of Rio Grande do Sul Animal Care and Use Committee (process number 21215). Briefly, the cerebral cortex of newborn Wistar rats (1–2 days old) was removed and mechanically dissociated in Ca^2+^- and Mg^2+^- free Hanks’ balanced salt solution (HBSS), pH 7.4, containing the following: 137 mM NaCl, 5.36 mM KCl, 0.27 mM Na_2_HPO_4_, 1.1 mM KH_2_PO_4_, and 6.1 mM glucose. The cortex was cleaned of the meninges and mechanically dissociated by sequential passage through a Pasteur pipette. After centrifugation at 1,000 rpm for 5 min, the pellet was resuspended in DMEM (pH 7.6) supplemented with 8.39 mM HEPES, 23.8 mM NaHCO_3_, 0.1% amphotericin B, 0.032% gentamicin and 10% FBS. Approximately 300,000 cells were seeded in each well of the 24-well plates and maintained in DMEM containing 10% FBS in 5% CO_2_/95% air at 37°C. The cells were allowed to grow to confluence and used after approximately 15 days *in vitro*.

### Treatments

At confluence, the culture medium was removed by suction, and the cells were treated with ammonia and antioxidants (resveratrol, L-NAME, ascorbic acid, trolox) at the indicated concentrations. Cells were also pre-treated for 1 h with resveratrol (100 µM), ascorbic acid (100 µM), trolox (50 µM) or L-NAME (500 µM) at 37°C in an atmosphere of 5% CO_2_/95% air in DMEM without serum. Subsequently, 5 mM ammonia (NH_4_Cl) was added in the presence or absence of resveratrol (100 µM), ascorbic acid (100 µM), trolox (50 µM) or L-NAME (500 µM) for 24 h at 37°C in an atmosphere of 5% CO_2_/95% air in DMEM without serum. For all parameters analyzed, the results obtained with vehicle were not different from those obtained under basal conditions.

To study the role of PKA and MEK/ERK pathways in the response of resveratrol against ammonia toxicity, we pre-treated the cells for 1 h with 10 µM H-89 and 5 µM PD98059, the specific PKA and MEK inhibitors, respectively. The role of NO metabolism was also evaluated using SNP, a NO donator (40 µM).

### Membrane Integrity and Metabolic Activity Assays

Propidium iodide incorporation assay: Cells were treated simultaneously with 7.5 µM PI and incubated for up to 24 h. The optical density of ﬂuorescent nuclei (labeled with PI), indicative of cell death, was determined with Optiquant software (Packard Instrument Company). Density values obtained are expressed as a percentage of the control values.

Lactate dehydrogenase assay: The lactate dehydrogenase (LDH) assay was conducted in 50 µL of extracellular medium using a commercial colorimetric assay from Doles (Brazil). Results are expressed as percentages of the control value.

MTT reduction assay: Cells were treated with 50 µg/mL MTT for 30 min in 5% CO_2_/95% air at 37°C. Subsequently, the medium was removed, and the MTT crystals were dissolved in DMSO. Absorbance values were measured at 560 and 650 nm. Results are expressed as percentages of the control value.

### DCFH Oxidation

Intracellular ROS production was detected using the non-fluorescent cell-permeating compound, 2′-7′-dichlorofluorescein diacetate (DCFH-DA). DCFH-DA is hydrolyzed by intracellular esterases to dichlorofluorescin (DCFH), which is trapped within the cell. This non-fluorescent molecule is then oxidized into fluorescent dichlorofluorescin (DCF) by the action of cellular oxidants. Cultured cells were treated as *described above. C*ells were then incubated with DCFH-DA (10 µM) for 30 min at 37°C. Following DCFH-DA exposure, the cells were scraped into PBS with 0.2% Triton X-100. The fluorescence was measured in a plate reader (Spectra Max GEMINI XPS, Molecular Devices, USA) with excitation at 485 nm and emission at 520 nm [38]. Results are expressed as percentages of the control value.

### Nitric Oxide Production

Nitric oxide was determined by measurement of nitrite (a stable oxidation product of NO), based on the Griess reaction. The Griess reagent was prepared by mixing equal volumes of 1% sulfanilamide in 0.5 N HCl and 0.1% N-(1-naphthyl) ethylenediamine in deionized water. The assay was performed as described [45] with modifications. Briefly, cells were cultured on 96-well plate and after treatment, the Griess reagent was added directly to the cell culture and the incubation was maintained under reduced light at room temperature during 15 min. Samples were analyzed at 550 nm on a microplate spectrophotometer. Controls and blanks were run simultaneously. Nitrite concentrations were calculated using a standard curve prepared with sodium nitrite (0–50 µM).

### Glutamine Synthetase Activity

The enzymatic assay was performed as previously described [27]. Briefly, homogenate was added to a reaction mixture containing 10 mM MgCl_2_, 50 mM L-glutamate, 100 mM imidazole-HCl buffer (pH 7.4), 10 mM 2-mercaptoethanol, 50 mM hydroxylamine–HCl and 10 mM ATP and incubated for 15 min at 37°C. The reaction was stopped by adding a solution containing 370 mM ferric chloride, 670 mM HCl and 200 mM trichloroacetic acid. After centrifugation, the absorbance of the supernatant was measured at 530 nm and compared to a calibration curve of γ-glutamylhydroxamate treated with ferric chloride reagent. Results are expressed as percentages of the control value.

### S100B Secretion Assay

S100B secretion was measured by an enzyme-linked immunosorbent assay, as previously described [46]. Briefly, 50 µL of sample and 50 µL of Tris buffer were incubated for 2 h on a microtiter plate previously coated with monoclonal anti-S100B (SH-B1). Next, the samples were incubated with polyclonal anti-S100B for 30 min, and then, peroxidase-conjugated anti-rabbit antibody was added for a further 30 min incubation period. A colorimetric reaction with *o*-phenylenediamine was observed at 492 nm. Results are expressed as percentages of the control value.

### Tumor Necrosis Factor α Measurement

TNF-α assay was carried out in extracellular medium, using a rat TNF-α ELISA from PeproTech (USA).

### Interleukins Measurement

IL-1β and IL-6 was carried out in cell culture supernatant, using the rat ELISA for IL-1β and IL-6, respectively, from eBioscience (USA).

### Nuclear Factor-κB Measurement

The levels of NF-κB p65 were measured using an ELISA commercial kit from Invitrogen (USA).

### Protein Determination

Protein content was measured by Lowry’s method using bovine serum albumin as the standard [47].

### Statistical Analysis

Data are presented as mean ± S.E.M. Each experiment was performed in triplicate from at least three independent cultures. The data were subjected to one/two-way analysis of variance (ANOVA) followed by the Tukeýs test. Values of P<0.05 were considered significant. All analyses were performed using the Statistical Package for the Social Sciences (SPSS) software.

## Results

The integrity and metabolic activity of C6 astroglial cells incubated with ammonia for 24 h, as evaluated by measuring PI incorporation, extracellular LDH content and MTT reduction (Table S1), did not significantly change relative to control conditions. The antioxidants resveratrol, L-NAME, ascorbic acid and trolox also did not produce significant changes in these cell viability parameters (Table S2), with the exception of 100 µM trolox. Morphological studies showed that ammonia exposure induced astroglial swelling and cell body retraction and resveratrol totally prevented this effect (Figure S1).

**Figure 1:**
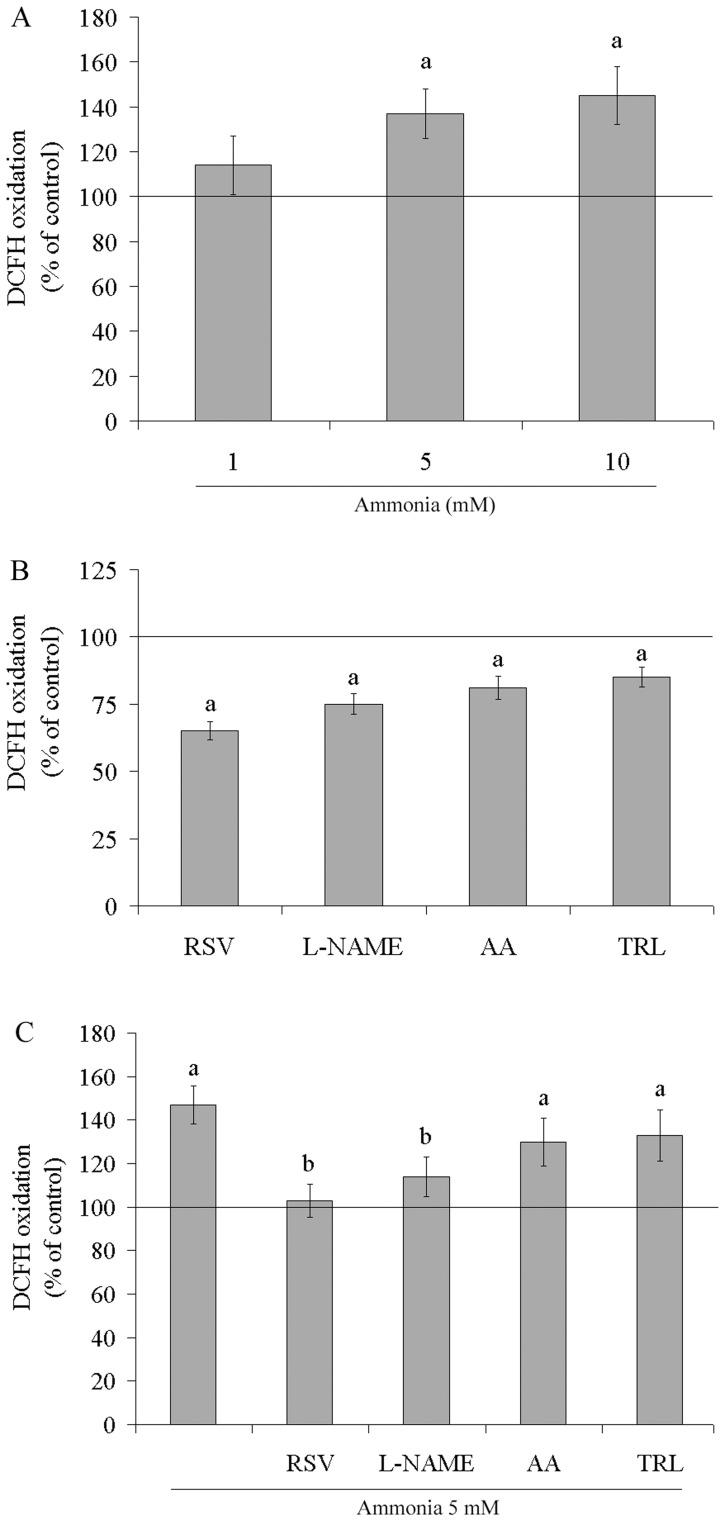
Effects of ammonia and antioxidants on DCFH oxidation. Cells were incubated with ammonia (1, 5 and 10 mM) – **A**, antioxidants (100 µM resveratrol (RSV), 500 µM L-NAME, 100 µM ascorbic acid (AA) and 50 µM trolox (TRL)) – **B**, or antioxidants plus ammonia (5 mM) – **C**, for 24 h. DCFH oxidation was measured as described in the Materials and methods section. The line indicates the control value. Data represent means ± S.E.M of three experimental determinations performed in triplicate, analyzed statistically by two-way ANOVA followed by the Tukey’s test. (a) indicates significant differences from the control (P<0.05). (b) indicates significant differences from ammonia (P<0.05).

**Figure 2:**
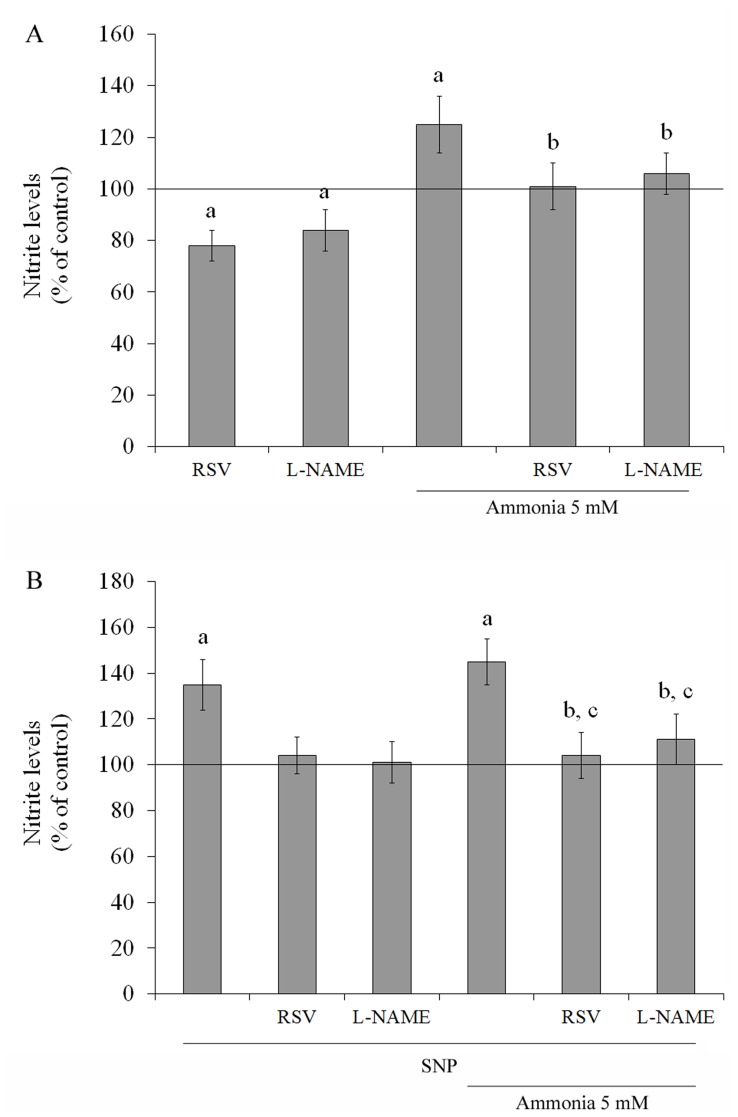
Effects of ammonia and antioxidants on NO levels. The production of NO was indirectly measured by the formation of nitrite. Cells were incubated for 24 h with 100 µM resveratrol (RSV) or 500 µM L-NAME in the presence or absence of 5 mM ammonia (**A**). Cells were treated with 40 µM SNP in the presence or absence of 100 µM resveratrol (RSV) or 500 µM L-NAME and 5 mM ammonia, for 24 h (**B**). Nitrite levels were measured as described in the Materials and methods section. The line indicates the control value. Data represent means ± S.E.M of three experimental determinations performed in triplicate, analyzed statistically by two-way ANOVA followed by the Tukey’s test. (a) Indicates significant differences from the control (P<0.05). (b) indicates significant differences from ammonia (P<0.05). (c) indicates significant differences from SNP (P<0.05).

The production of ROS was monitored using DCFH oxidation. The level of DCFH oxidation in C6 astroglial cells increased by about 40% and 55% following treatment for 24 h with 5 and 10 mM ammonia, respectively, indicating an increase in ROS production (Figure 1A). All of the antioxidants tested decreased the basal level of DCFH oxidation (Figure 1B). Resveratrol was the most active (35%), followed by L-NAME (25%), trolox (19%) and ascorbic acid (15%). Next, we evaluated the effect of antioxidants on ROS production in the presence of ammonia (Figure 1C). Resveratrol and L-NAME successfully protected the cells; the DCFH oxidation levels dropped from 147±8.7% to 103±7.6% and 114±9%, respectively. The production of NO was indirectly measured by the formation of nitrite (Figure 2A). Both resveratrol and L-NAME decreased nitrite production, 22% and 16%, respectively, compared to the control conditions. Ammonia increased nitrite formation up to 25%. This effect was completely prevented by resveratrol and L-NAME. SNP, a NO donator, was used as a positive control for NO production (Figure 2B). SNP (40 µM –24 h) increased by about 35% the nitrite levels compared to basal cultures. Ammonia plus SNP also increased nitrite levels (45%). Resveratrol and L-NAME totally prevent this effect, restoring levels to control conditions.

**Figure 3:**
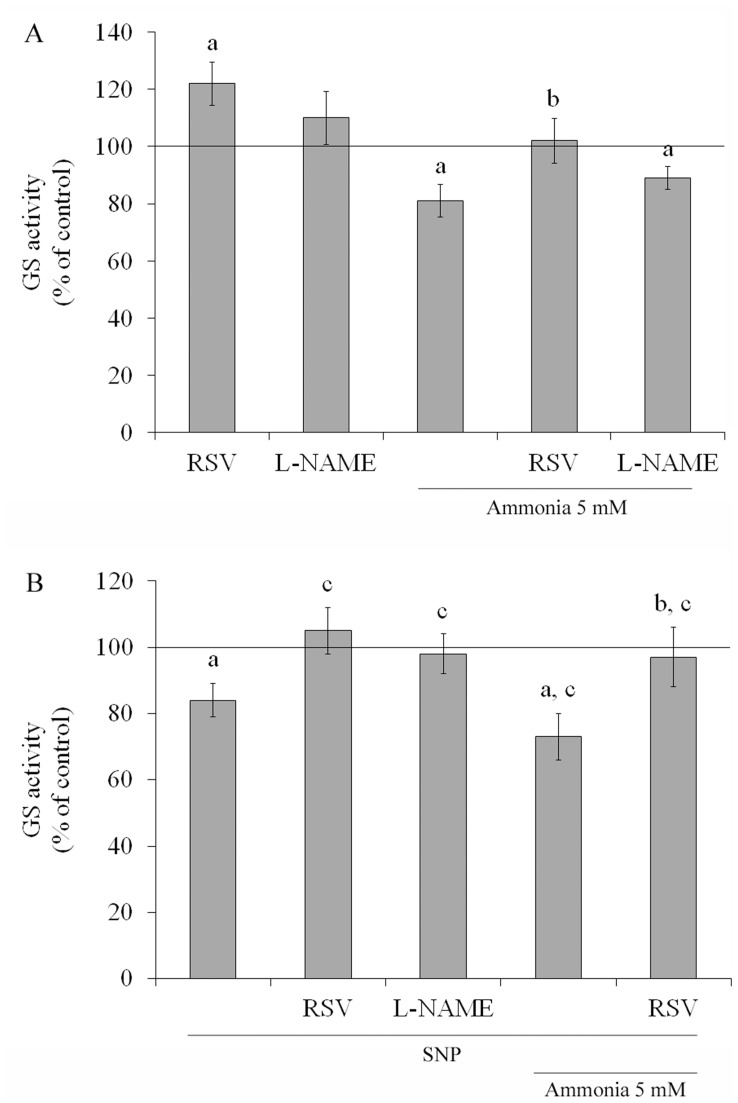
Effects of ammonia and antioxidants on GS activity. Cells were incubated for 24 h with 100 µM resveratrol (RSV) or 500 µM L-NAME in the presence or absence of ammonia (5 mM) – **A**. Cells were treated with 40 µM SNP in the presence or absence of 100 µM resveratrol (RSV) or 500 µM L-NAME and 5 mM ammonia, for 24 h – **B**. GS activity was measured as described in the Materials and methods section. The line indicates the control value. Data represent means ± S.E.M of three experimental determinations performed in triplicate, analyzed statistically by two-way ANOVA followed by the Tukey’s test. (a) Indicates significant differences from the control (P<0.05). (b) indicates significant differences from ammonia (P<0.05). (c) indicates significant differences from SNP (P<0.05).

Because GS is primarily responsible for clearing ammonia in the CNS, we measured the GS activity of C6 astroglial cells following exposure to ammonia (Figure 3A). GS activity decreased by about 19% compared to control conditions after exposure for 24 h to 5 mM ammonia. Because resveratrol and L-NAME effectively prevented the induction of increased ROS production by ammonia, we evaluated their effects on GS activity. Resveratrol *per se* increased the activity of GS, by about 20% and completely prevented the ammonia-induced decrease in GS activity (81±5.7% to 102±7.9%), but L-NAME did not.

SNP was used indirectly to investigate whether the effect of resveratrol on GS activity was mediated by NO (Figure 3B). SNP decreased GS activity (16%) and also potentiated the decreased on GS activity induced by ammonia (27%). Resveratrol co-incubated with SNP and under ammonia exposure was able to prevent the decrease. As expected, L-NAME blocked the effect of SNP on GS activity. These data indicate that nitrosative stress and nitric oxide synthase (NOS) activity can mediate the effect of resveratrol on GS activity.

**Figure 4:**
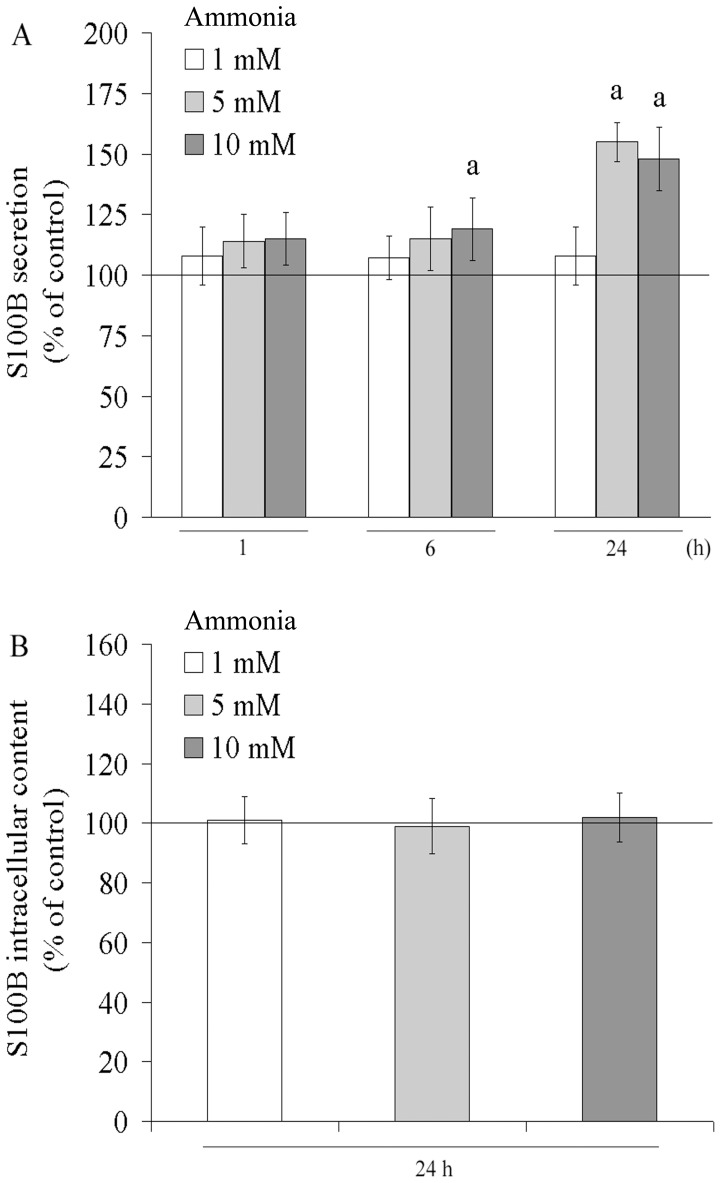
Effect of ammonia on S100B secretion in C6 astroglial cells. Cells were incubated for 1, 6 and 24 h with ammonia (1, 5 and 10 mM). S100B extracellular (**A**) and intracellular (**B**) content was measured as described in the Materials and methods section. The basal secretion level, assumed to be 100%, is indicated by the line. Data represent means ± S.E.M of three experimental determinations performed in triplicate, analyzed statistically by one-way ANOVA followed by the Tukey’s test. (a) indicates significant differences from the control (P<0.05).

The secretion of S100B by C6 astroglial cells was not affected by incubation with 1, 5 or 10 mM of ammonia for 1 h (Figure 4A). After 6 h of treatment with 10 mM of ammonia, however, S100B secretion increased by about 20%. Following 24 h of exposure to ammonia, the level of extracellular S100B increased significantly, by about 55% and 48% with 5 mM and 10 mM of ammonia, respectively. Because changes in cell integrity were not observed, the increased levels of extracellular S100B most likely resulted from secretion. The intracellular S100B content, however, was not affected by ammonia (Figure 4B). This result may indicate glial activation because S100B was released without being over-expressed.

**Figure 5:**
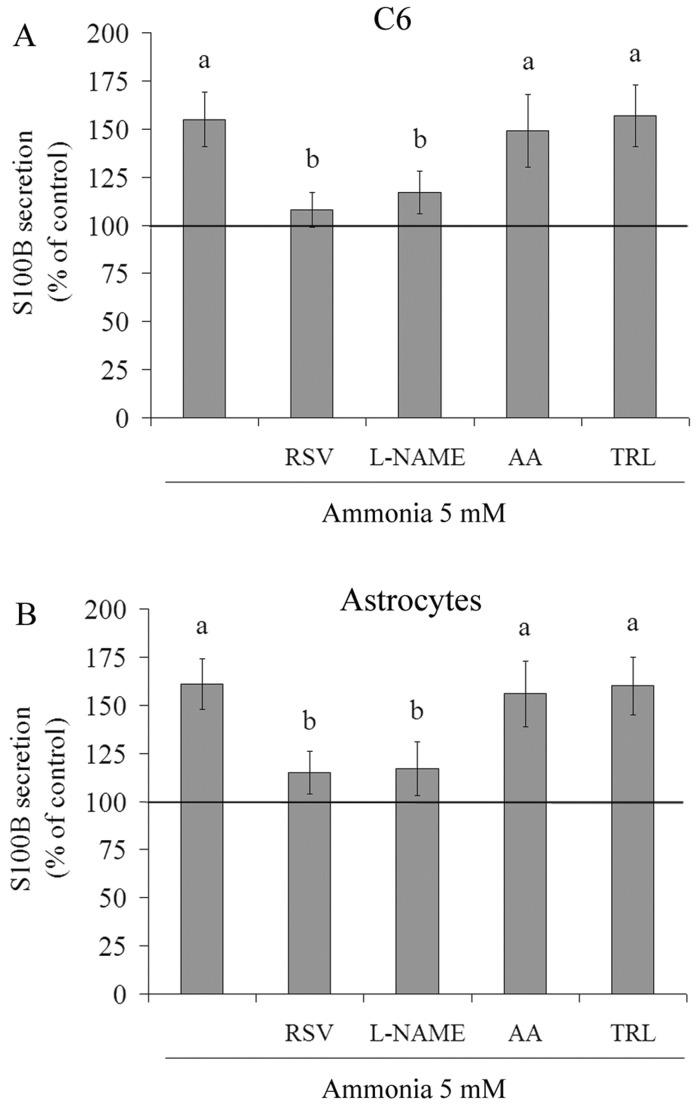
Effect of antioxidants on ammonia-induced S100B secretion in C6 astroglial cells and primary astrocyte cultures. C6 astroglial cells (**A**) and primary astrocytes (**B**) were pre-treated for 1 h with 100 µM resveratrol (RSV), 500 µM L-NAME, 100 µM ascorbic acid (AA) or 50 µM trolox (TRL). After pre-treatment, ammonia (5 mM) was added in the presence or absence of these antioxidants. The basal secretion level, assumed to be 100%, is indicated by the line. Data represent means ± S.E.M of three experimental determinations performed in triplicate, analyzed statistically by two-way ANOVA followed by the Tukey’s test. (a) indicates significant differences from the control (P<0.05). (b) indicates significant differences from ammonia (P<0.05).

Under ammonia-induced oxidative stress, resveratrol decreased S100B secretion from 155±14% to 108±9%, relative to ammonia *per se* (Figure 5A). L-NAME produced a similar effect. Ascorbic acid and trolox maintained or did not significantly reduce the level of ammonia-induced S100B secretion. As C6 cells and primary astrocytes have shown contrasting profile of *in vitro* S100B secretion [48], here we also examined the effect of ammonia and antioxidants in primary astrocytes. Both stimuli produced similar effects on S100B secretion by primary astrocytes (Figure 5B), confirming in our study the similar responses between these cells. It is important to mention that antioxidants *per se* did not affect the levels of extracellular S100B in primary astrocytes (data not shown).

**Figure 6:**
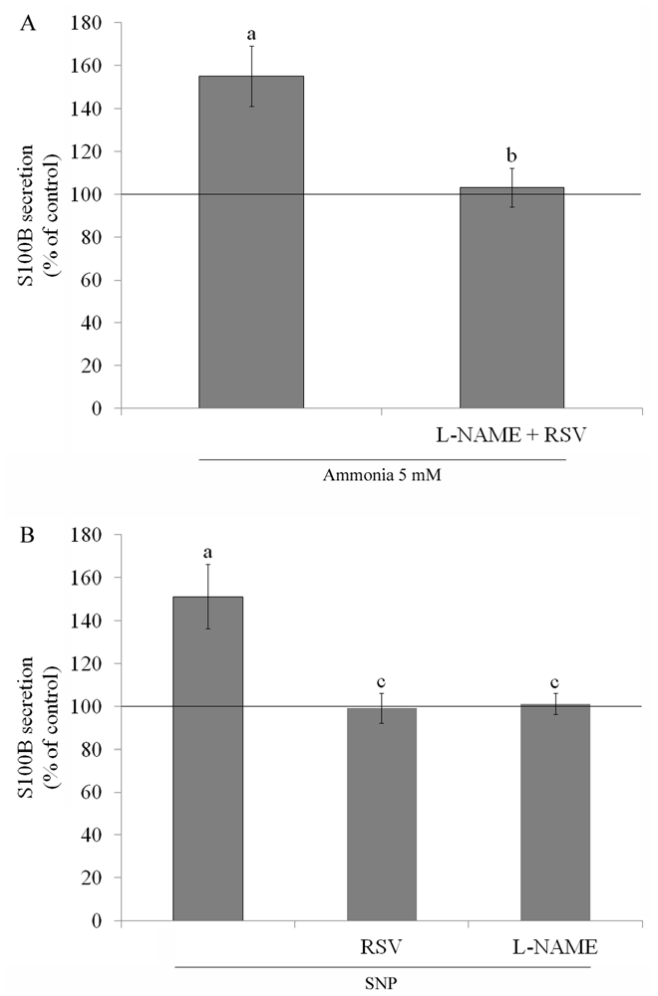
Effect of simultaneous resveratrol and L-NAME treatment and SNP on ammonia-induced S100B secretion in C6 astroglial cells. Cells were pre-treated with 100 µM resveratrol (RSV) and 500 µM L-NAME simultaneously, in the presence of 5 mM ammonia for 24 h (**A**)**.** Cells were pre-treated for 1 h with 100 µM resveratrol (RSV) or 500 µM L-NAME, followed by incubation with 40 µM SNP for 24 h (**B**). The basal secretion level, assumed to be 100%, is indicated by the line. Data represent means ± S.E.M of three experimental determinations performed in triplicate, analyzed statistically by two-way ANOVA followed by the Tukey’s test. (a) indicates significant differences from the control (P<0.05). (b) indicates significant differences from ammonia (P<0.05). (c) indicates significant differences from SNP (P<0.05).

Simultaneous addition of resveratrol and L-NAME reduced S100B in C6 astroglial cells by about 55%, compared to ammonia alone (Figure 6A). SNP was used to examine whether alterations on S100B secretion was dependent of NO production (Figure 6B). SNP increased 51% the S100B release. Resveratrol and L-NAME restore the levels to near the basal values, indicating that the increase in S100B is related to NO metabolism.

**Figure 7:**
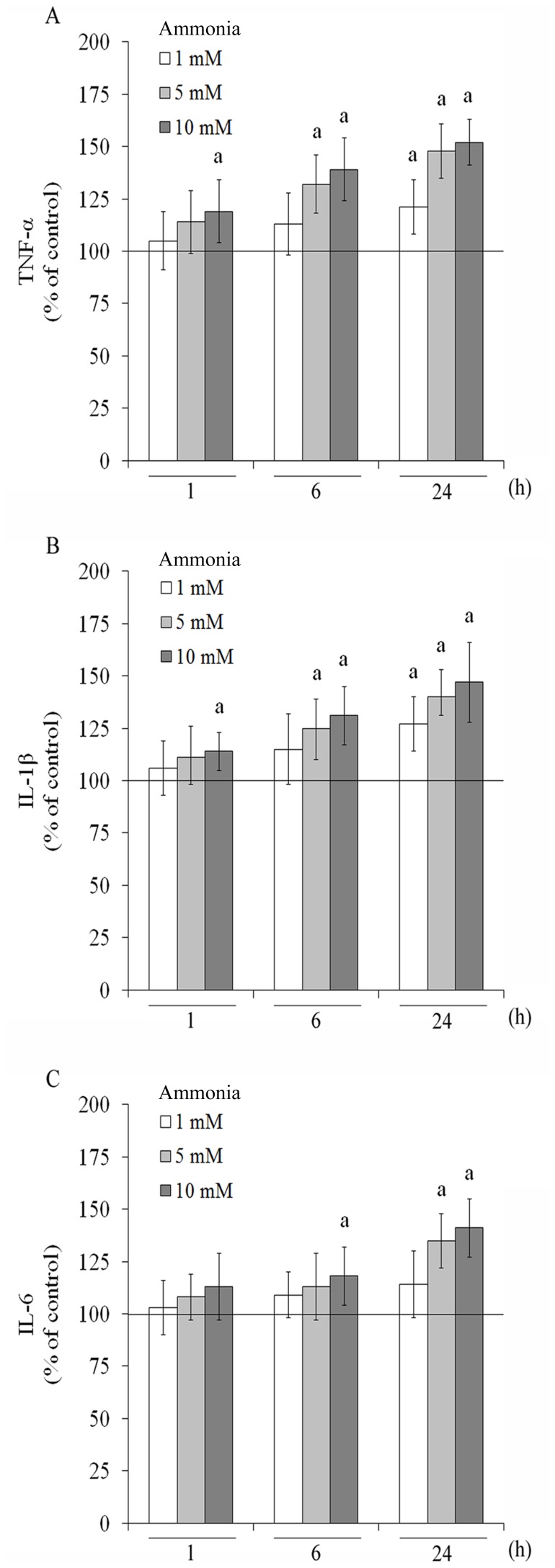
Effect of ammonia on cytokines release in C6 astroglial cells. Cells were incubated for 1, 6 and 24 h with ammonia (1, 5 and 10 mM). TNF-α (**A**), IL-1β (**B**) and IL-6 (**C**) levels were measured as described in the Materials and methods section. The basal cytokines levels, assumed to be 100%, is indicated by the line. Data represent means ± S.E.M of three experimental determinations performed in triplicate, analyzed statistically by one-way ANOVA followed by the Tukey’s test. (a) indicates significant differences from the control (P<0.05).

ROS play a critical role in inflammatory response. Moreover, neuroinflammation has been described in a wide variety of neurological disorders, including HE. In this sense, we evaluated the classical proinflammatory cytokine, TNF-α (Figure 7A). After 1 h of exposure, ammonia 10 mM increased TNF-α release by about 19%. At 6 h, 5 and 10 mM ammonia increased TNF-α release by about 32% and 39%, compared to control conditions, respectively. Following 24 h of exposure to ammonia, all concentrations increased TNF-α levels. IL-1β, another proinflammatory cytokine, was also measured (Figure 7B) and a significant increase was observed at 1 h of 10 mM ammonia exposure. An increase of 25% and 31% in IL-1β levels was observed with 5 and 10 mM of ammonia, respectively, after 6 h exposure. IL-1β increased from 1 mM ammonia and higher concentrations at 24 h of exposure. Ammonia also modulated positively the release of IL-6 (Figure 7C). At 6h, 10 mM ammonia increased 18% IL-6 levels, compared to control conditions. After 24 h, 5 and 10 mM of ammonia induced a significant increase (by about 35% and 41%, respectively) in the cytokine IL-6.

**Figure 8:**
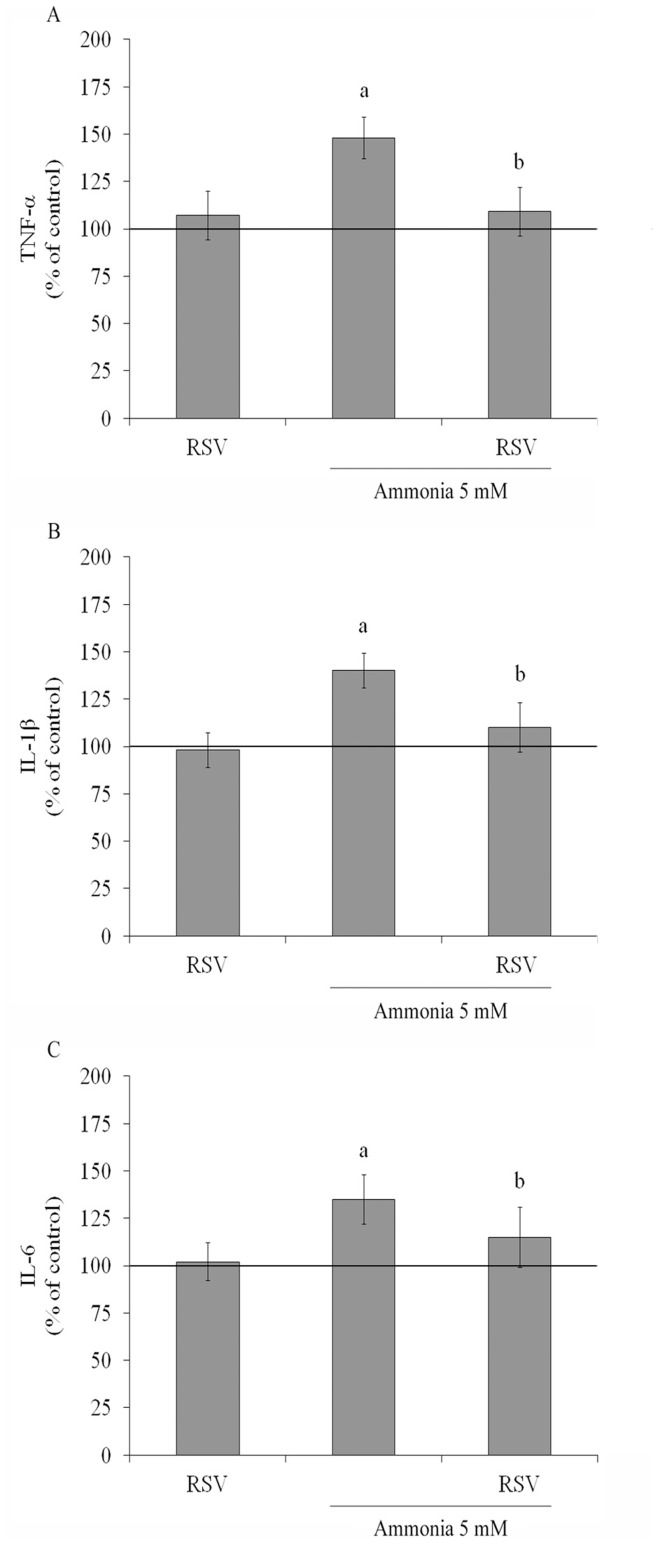
Effect of resveratrol on ammonia-induced cytokines in C6 astroglial cells. Cells were pre-treated for 1 h with 100 µM resveratrol (RSV). After pre-treatment, ammonia (5 mM) was added in the presence or absence of resveratrol. The basal cytokines levels, assumed to be 100%, is indicated by the line. Data represent means ± S.E.M of three experimental determinations performed in triplicate, analyzed statistically by two-way ANOVA followed by the Tukey’s test. (a) indicates significant differences from the control (P<0.05). (b) indicates significant differences from ammonia (P<0.05).

To determine whether resveratrol could modulate the ammonia-stimulated proinflammatory cytokines, we evaluated the levels of TNF-α, IL-1β and IL-6 in C6 astroglial cells. Resveratrol *per se* did not affect the levels of these cytokines, but is able to completely prevent the augment induced by ammonia exposure, decreasing TNF-α levels from 148±13% to 109±13% (Figure 8A); IL-1β levels from 140±9% to 110±13% (Figure 8B) and IL-6 from 135±13% to 115±16% (Figure 8C), values expressed as percentage of control.

**Figure 9:**
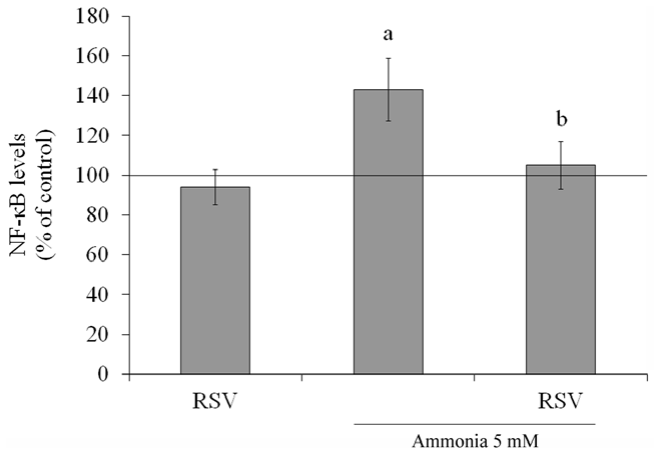
Effect of resveratrol on NF-κB levels in C6 astroglial cells. Cells were pre-treated for 1 h with 100 µM resveratrol (RSV) followed by incubation with 5 mM ammonia for 24 h. The basal NF-κB level, assumed to be 100%, is indicated by the line. Data represent means ± S.E.M of three experimental determinations performed in triplicate, analyzed statistically by two-way ANOVA followed by the Tukey’s test. (a) indicates significant differences from the control (P<0.05). (b) indicates significant differences from ammonia (P<0.05).

NF-κB is a transcription factor that induces the expression of genes involved in many biological responses including inflammation [49]. Ammonia increased the NF-κB levels by about 43% (Figure 9). Resveratrol alone did not significantly change the levels, but it was able to prevent the increase induced by ammonia exposure from 143±16% to 105±12%. As expected, H-89 and PD98059 inhibited the NF-κB translocation (data not shown).

The effect of ammonia on cytokines release can involve many pathways, one them involves cAMP. In fact, cAMP mediate ammonia toxicity [50], [51] and we investigated whether PKA was involved in the increase of S100B secretion and proinflammatory cytokines provoked by ammonia in C6 astroglial cells (Table 1). Cells were pre-treated with a specific PKA inhibitor, H-89 (10 µM), and then incubated with ammonia. The PKA inhibitor prevented the increase in S100B release after 24 h of incubation with ammonia. H-89 also prevented the increase in ammonia-stimulated proinflammatory cytokines. The co-incubation with resveratrol, ammonia and H-89 did not show results different from ammonia and H-89, indicating that resveratrol probably does not act directly by PKA.

**Table 1:** PKA pathway modulates ammonia-induced cytokines release.

Treatments	S100B	TNF-α	IL-1β	IL-6
Ammonia	155±14 (a)	148±13 (a)	140±9 (a)	135±13 (a)
H-89	106±10	100±8	101±8	93±13
Ammonia+H-89	108±9 (b)	109±11 (b)	113±15 (b)	120±11 (b)
RSV+H-89	105±9 (b)	104±9 (b)	103±11 (b)	109±11 (b)
Ammonia+RSV+H89	99±6 (b)	101±10 (b)	104±4 (b)	105±8 (b)

C6 astroglial cells were pre-incubated with 10 µM PKA inhibitor (H-89), followed by treatment with 100 µM resveratrol (RSV) and 5 mM ammonia for 24 h. Data are expressed as percentage of control values and represent means ± S.E.M of three experimental determinations performed in triplicate, analyzed statistically by two-way ANOVA followed by the Tukey’s test. (a) indicates significant differences from the control (P<0.05). (b) indicates significant differences from ammonia (P<0.05).

ERK pathway has also been implicated in the regulation of glial inflammatory responses following an insult [52]. In this sense, we examined the S100B and proinflammatory cytokines release using a MEK/ERK inhibitor, PD98059 (Table 2). Under ammonia exposure, MEK/ERK inhibitor prevented the increase in all cytokines. Moreover, resveratrol potentiated the effect of PD98059 in astroglial cells, decreasing the release of S100B, TNF-α, IL-1β and IL-6 lower that ammonia plus PD98059. These results indicated the involvement of ERK signaling in the protective mechanism of resveratrol against hyperammonemia.

**Table 2:** Resveratrol protects ammonia-induced cytokines release through ERK signaling pathway.

Treatments	S100B	TNF-α	IL-1β	IL-6
Ammonia	153±13 (a)	148±13 (a)	140±9 (a)	135±13 (a)
PD	89±9 (b)	103±10 (b)	102±11 (b)	93±10 (b)
Ammonia+PD	115±9 (a,b,c)	109±10 (b)	108±12 (b)	109±9 (b)
RSV+PD	72±5 (a,b,c)	88±11 (b,c)	87±9 (b,c)	80±7 (a,b,c)
Ammonia+RSV+PD	87±7 (a,b)	85±9 (a,b,c)	81±10 (a,b,c)	83±9 (a,b,c)

C6 astroglial cells were incubated with 5 µM MEK/ERK inhibitor (PD98059– referred in the table as PD), followed by treatment with 100 µM resveratrol (RSV) and 5 mM ammonia for 24 h. Data are expressed as percentage of control values and represent means ± S.E.M of three experimental determinations performed in triplicate, analyzed statistically by two-way ANOVA followed by the Tukey’s test. (a) indicates significant differences from the control (P<0.05). (b) indicates significant differences from ammonia (P<0.05). (c) indicates significant differences from MEK/ERK inhibitor (P<0.05).

## Discussion

Oxidative stress has been implicated in ammonia neurotoxicity [10]. In the brain *in vivo* and in cultured cells, this condition results in diminished antioxidant defense activity [53], [54]. In this study, we observed that treatment of C6 astroglial cells with ammonia increased the level of DCFH oxidation and nitrite production. Previously, *Haghighat et al.* [55] showed that ammonia interfered with ATP production, leading to reduced levels of NADH and increased NO levels, with subsequent nitration/inactivation of enzymes [56]. We found that resveratrol and L-NAME, two important antioxidants with many biological effects, prevented the ammonia-induced increase of ROS/RNS production. This effect may arise from improved antioxidant defense and/or from inhibition of NOS.

In the CNS, ammonia is primarily detoxified in astrocytes by the enzyme GS, which catalyses the ATP-dependent amidation of glutamate to form glutamine. Continuing the glutamate-glutamine cycle, the glutamine thus formed is exported to neurons, allowing the synthesis of glutamate [57], [58]. GS is very sensitive to oxidative and nitrosative stress. Consistent with the results of *Leite et al.* [16], we have demonstrated that ammonia toxicity decreases GS activity in astroglial cells. Moreover, the NO metabolism seems to be related to GS activity failure (Figure 3). Resveratrol acted as scavenger more efficiently than L-NAME. Previously, we have demonstrated that resveratrol *per se* increases values of glial function parameters such as glutamate uptake, GS activity and glutathione content under oxidative stress [27], [36], [38], [39]. Recent reports showed that HO1 (heme oxygenase 1) and its transcription factor regulator, nuclear factor erythroid-2 related factor-2 (Nrf2), might control the neuroprotective effect of resveratrol [59], [60]. Resveratrol may therefore be effective against ammonia toxicity either by its ROS/RNS-scavenging effect directly and/or by inhibiting iNOS, a protein downstream of HO1 (Quincozes-Santos, manuscript in revision) [61]. Additionally, the increase in the activity of GS could be related to increased production of glutathione, the main antioxidant of the CNS [22], [62], [63], [64]. It is important to mention that Nrf2 also facilitates the GSH synthesis [65].

During this study, we also investigated the effect of ammonia on the release of S100B from astroglial cells. Elevated serum levels of S100B were observed in HE [66], and increased levels of S100B secretion were also observed in astrocytes exposed to ammonia [16]. We verified that S100B secretion was stimulated by ammonia in the C6 astrocyte cell line. This effect cannot be attributed to astroglial cell death because cell viability was not altered by ammonia. It is widely known that S100B secretion is affected by redox conditions and metabolic stress [38], [48], [67], [68], [69]. Increases in extracellular S100B concentrations may be related to brain damage/glial reactivity, and persistent high levels could be involved in neurodegenerative disorders [14]. We observed that increased S100B release was accompanied by increased ROS production and decreased GS activity, indicating a glial response. These effects may be associated with the impairment of several cellular functions.

Hyperammonemia in glial cells results in increased cellular levels of calcium and cAMP [3], [9], [51], [70]. Both calcium and cAMP have been proposed to mediate S100B release [71], [72]. Treatment with the PKA inhibitor H-89 completely prevented the ammonia-induced increase of extracellular S100B levels. Moreover, ammonia induces ERK activation [13] and the effect of S100B release was totally abolished by MEK/ERK inhibitor, indicating that cAMP/PKA and ERK are involved in this effect.

When astroglial cells were incubated with antioxidants and ammonia, only resveratrol and L-NAME (an NOS-inhibitor) protected against ammonia toxicity. Data on resveratrol and L-NAME indicate that NO and/or its toxic derivative peroxynitrite are probably involved in these effects. Co-incubation with resveratrol and L-NAME produced a reduction in S100B secretion, probably indicating an interaction between the two compounds. We observed that SNP, a NO donator, stimulated the S100B secretion, and resveratrol and L-NAME were able to prevent this effect, indicating that both could act in the NO pathway. Then, the mechanism by which resveratrol modulates S100B secretion may therefore involve NO. It is important to point out that the mechanisms of S100B secretion and action of resveratrol are not well defined.

Ammonia also increased the main proinflammatory cytokines: TNF-α and IL-1β. These cytokines have a major role in initiating a cascade of activation of other cytokines and growth factor in inflammatory response [73]. TNF-α is synthesized mainly by microglia and astrocytes, and has several important functions in the CNS, including astrocytes activation and glutamatergic gliotransmission [30], [73], [74]. Recent studies show that proinflammatory cytokines are increased in HE [75], [76], may be used as a marker for encephalopathy grade. TNF-α and IL-1β acts since acute inflammatory responses and both induces IL-6 synthesis [73]. IL-6 is also produced in microglia, astrocytes and neurons, and it plays a pivotal role in a variety of CNS functions such as induction and modulation of astrocytes reactivation, pathological inflammatory and neuroprotection. Assuming S100B as a cytokine, it present the same profile of augment IL-6 and different of other cytokines their present a duality of actions: degenerative and reparative and/or anti and proinflammatory [77], [78], [79]. Moreover, the negative effects of these cytokines may be mediated by TNF-α and IL-1β. Interestingly, as well as the effect on S100B was via PKA and ERK, the increases of other cytokines were also cAMP/PKA/ERK-dependent pathways.

The mechanism of NF-κB activation and subsequent translocation into the nucleus involves a variety of signaling pathways and modifications, which are not completely understood [80]. A significant increase in NF-κB levels was observed after treatment of C6 cells with ammonia. NF-κB is a transcription factor involved in immune and inflammatory reactions [81], which was inhibited by resveratrol. This regulation could explain the decrease in cytokines release. Besides, PKA and ERK signaling pathway can activate NF-κB [41], [49]. Resveratrol potentiated the effect of MEK/ERK inhibitor and this data may provide a mechanistic explanation for the protective effect of resveratrol against inflammatory cytotoxicity induced by ammonia. NF-κB also induces the expression of iNOS [82]. Thus, we demonstrated that resveratrol decreased S100B-ammonia induced through ERK signaling, inhibition of NF-κB and NO metabolism.

TNF-α plays important roles in ROS production [83] and it also potentiates NO production in astrocytes [84]. Thus, an extracellular stimulus such as ammonia induces an increase in ROS/RNS production and in four cytokines, which are related to redox environment; therefore, the augment may also be through oxidative/nitrosative stress. In this sense, we observed that resveratrol was able to prevent the cytokines increase induced by ammonia exposure, probably through ERK signaling pathway, an upstream signal transduction of NF-κB. Ammonia induces, in addition to overproduction of ROS, the protein tyrosine nitration and oxidation of RNA [85], [86]. Our group recently demonstrated that resveratrol prevented astroglial cells against H_2_O_2_-induced genotoxicity [26], [28]. Even though its mechanism of action remains a mystery, resveratrol may exert its effects by antioxidant/scavenger activity, by modulated NO metabolism or by anti-inflammatory effect [32], [34], [87], [88]. As well, clinical studies showed that N-acetylcysteine (NAC), an antioxidant precursor of glutathione, present protection averts neuroinflammation due to HE [75].

**Figure 10:**
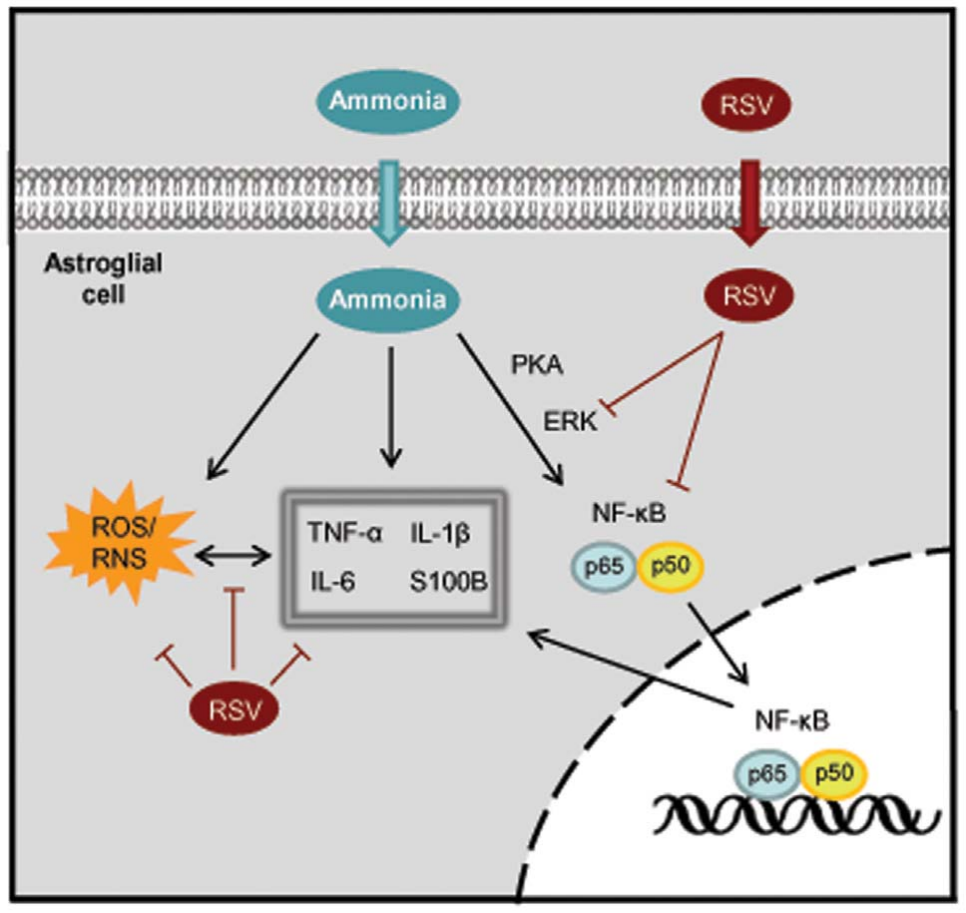
Proposed schematics of ammonia-induced cytokines and resveratrol protection in astroglial cells. Ammonia induces increase in intracellular ROS production and cytokines, probably through cAMP/PKA, ERK and NO. Resveratrol (RSV) decreases the levels of ROS and cytokines. The protective mechanism by which resveratrol prevents cytokines production is through inhibition of ERK signaling pathway and NF-κB and reduction of NO levels. Thus, RSV protects astroglial cells against ammonia-induced oxidative stress and/or cytokines release.

Our results suggest, for the first time, that resveratrol prevents ammonia toxicity in astroglial cells by attenuating oxidative and nitrosative stress, GS activity and cytokines release. Treatment of C6 astroglial cells with ammonia induced increases in S100B, TNF-α, IL-1β and IL-6 after 24 h of exposure via a mechanism dependent upon PKA, ERK and NO. Resveratrol prevented ammonia-induced cytokines release through inhibition of ERK signaling pathway and NF-κB and reduction of NO levels. The main conclusions about cytokines in this study are depicted in Figure 10. Overall, these observations suggest that resveratrol may potentially be useful for protection against oxidative stress and inflammatory response in ammonia-induced gliopathy. However, further studies are necessary to investigate the neuroprotective effects of resveratrol against *in vivo* hyperammonemia in both animal models and clinical trials.

## Supporting Information

Figure S1 **C6 astroglial cells morphology.** Cells were incubated for 24 h with 5 mM ammonia in the presence or absence of 100 µM resveratrol (RSV). Under normal conditions (Basal), the cells present polygonal morphology as shown by phase contrast microscopy. Ammonia induced astrocyte swelling and body retraction and RSV prevents this effect. Representative images of three experiments performed in triplicate.
(TIF)

Table S1 **Effect of ammonia on membrane integrity and metabolic activity in C6 astroglial cells.** C6 astroglial cells were incubated with ammonia (1, 5 and 10 mM) for 24 h. Membrane integrity and metabolic activity were measured as described in the Materials and methods section. Data are expressed as percentage of control values and represent means ± S.E.M of three experimental determinations performed in triplicate, analyzed statistically by one-way ANOVA followed by the Tukey’s test.
(DOCX)

Table S2 **Effect of antioxidants on membrane integrity and metabolic activity in astroglial cells.** C6 astroglial cells were incubated for 24 h in the presence of antioxidants - resveratrol (RSV), L-NAME, ascorbic acid (AA) and trolox (TRL) - at the indicated concentrations. Membrane integrity and metabolic activity were measured as described in the Materials and methods section. Data are expressed as percentage of control values and represent means ± S.E.M of three experimental determinations performed in triplicate, analyzed statistically by one-way ANOVA followed by the Tukey’s test. (a) indicates significant differences from the control (P<0.05).
(DOCX)
