# Supplementary figures and images for: Resveratrol Prevents Ammonia Toxicity in Astroglial Cells
Source: PLoS One. 2012 Dec 21;7(12):e52164. doi: 10.1371/journal.pone.0052164 (PMC3528750; doi:10.1371/journal.pone.0052164)

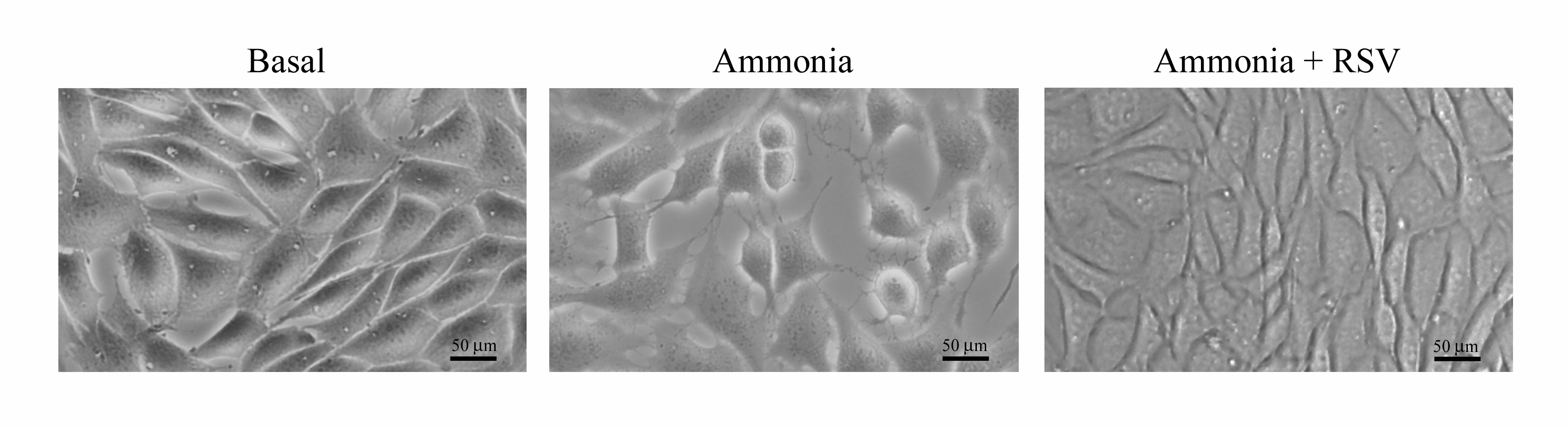

Supplement: Figure S1 — C6 astroglial cells morphology. Cells were incubated for 24 h with 5 mM ammonia in the presence or absence of 100 µM resveratrol (RSV). Under normal conditions (Basal), the cells present polygonal morphology as shown by phase contrast microscopy. Ammonia induced astrocyte swelling and body retraction and RSV prevents this effect. Representative images of three experiments performed in triplicate. (TIF) [file pone.0052164.s001.tif]
