# Supplementary material for: Resveratrol Prevents Ammonia Toxicity in Astroglial Cells
Source: PLoS One. 2012 Dec 21;7(12):e52164. doi: 10.1371/journal.pone.0052164 (PMC3528750; doi:10.1371/journal.pone.0052164)
Supplement: Table S1 — Effect of ammonia on membrane integrity and metabolic activity in C6 astroglial cells. C6 astroglial cells were incubated with ammonia (1, 5 and 10 mM) for 24 h. Membrane integrity and metabolic activity were measured as described in the Materials and methods section. Data are expressed as percentage of control values and represent means ± S.E.M of three experimental determinations performed in triplicate, analyzed statistically by one-way ANOVA followed by the Tukey’s test. (DOCX) [file pone.0052164.s002.docx]

| Ammonia (mM) | PI | LDH | MTT |
| --- | --- | --- | --- |
| 1 | 99 ± 5 | 100 ± 12 | 97 ± 5 |
| 5 | 98 ± 5 | 96 ± 11 | 96 ± 4 |
| 10 | 98 ± 5 | 97 ± 12 | 95 ± 6 |

**Table S1. Effect of ammonia on membrane integrity and metabolic activity in C6 astroglial cells.**

C6 astroglial cells were incubated with ammonia (1, 5 and 10 mM) for 24 h. Membrane integrity and metabolic activity were measured as described in the Materials and methods section. Data are expressed as percentage of control values and represent means ± S.E.M of three experimental determinations performed in triplicate, analyzed statistically by one-way ANOVA followed by the Tukey’s test.
