# Supplementary material for: Resveratrol Prevents Ammonia Toxicity in Astroglial Cells
Source: PLoS One. 2012 Dec 21;7(12):e52164. doi: 10.1371/journal.pone.0052164 (PMC3528750; doi:10.1371/journal.pone.0052164)
Supplement: Table S2 — Effect of antioxidants on membrane integrity and metabolic activity in astroglial cells. C6 astroglial cells were incubated for 24 h in the presence of antioxidants - resveratrol (RSV), L-NAME, ascorbic acid (AA) and trolox (TRL) - at the indicated concentrations. Membrane integrity and metabolic activity were measured as described in the Materials and methods section. Data are expressed as percentage of control values and represent means ± S.E.M of three experimental determinations performed in triplicate, analyzed statistically by one-way ANOVA followed by the Tukey’s test. (a) indicates significant differences from the control (P<0.05). (DOCX) [file pone.0052164.s003.docx]

|  | **C6** | | | **Astrocytes** | | |
| --- | --- | --- | --- | --- | --- | --- |
| **Treatments** | **PI** | **LDH** | **MTT** | **PI** | **LDH** | **MTT** |
| RSV (100 µM) | 102 ± 5 | 102 ± 12 | 110 ± 4 | 100 ± 5 | 100 ± 13 | 108 ± 5 |
| L-NAME (500 µM) | 100 ± 5 | 97 ± 11 | 107 ± 5 | 101 ± 5 | 96 ± 11 | 103 ± 6 |
| AA (100 µM) | 101 ± 5 | 95 ± 13 | 102 ± 4 | 98 ± 5 | 97 ± 12 | 101 ± 6 |
| AA (500 µM) | 104 ± 5 | 95 ± 12 | 99 ± 5 | 94 ± 5 | 95 ± 11 | 98 ± 5 |
| TRL (50 µM) | 103 ± 5 | 104 ± 10 | 98 ± 5 | 97 ± 4 | 95 ± 10 | 98 ± 7 |
| TRL (100 µM) | 117± 5 (a) | 112 ± 10 | 89 ± 5 (a) | 112 ± 5 (a) | 107 ± 11 | 90 ± 5 (a) |

**Table S2. Effect of antioxidants on membrane integrity and metabolic activity in astroglial cells.**

C6 astroglial cells were incubated for 24 h in the presence of antioxidants - resveratrol (RSV), L-NAME, ascorbic acid (AA) and trolox (TRL) - at the indicated concentrations. Membrane integrity and metabolic activity were measured as described in the Materials and methods section. Data are expressed as percentage of control values and represent means ± S.E.M of three experimental determinations performed in triplicate, analyzed statistically by one-way ANOVA followed by the Tukey’s test. (a) indicates significant differences from the control (P < 0.05).
